# Supplementary material for: Left behind in primary healthcare: A qualitative exploration of healthcare experiences of people with disabilities in Ethiopia
Source: PLOS Glob Public Health. 2025 Sep 26;5(9):e0005147. doi: 10.1371/journal.pgph.0005147 (PMC12469150; doi:10.1371/journal.pgph.0005147)
Supplement: S1 Text — (DOCX) [file pgph.0005147.s001.docx]

**Interview Guide – to explore healthcare experiences of people with disabilities**

**Induction**: -

1. Begin with a **greeting** & identify yourself by name and organization.
2. Remind the interviewee of the **aim** of the interview/ study
3. Ensure the interviewee understands their rights, including confidentiality and consent.
4. Remind them that they are **free** to decline to answer any of the questions or stop the interview at any time.
5. Check if they have any **questions** before you start.
6. Start recording

**Part I: Socio-demographic characteristics**

| **To be filled for each participant** | |
| --- | --- |
| Interview code |  |
| Interview mode (e.g., phone, audio, face to face, email etc.) |  |
| Interview date |  |
| Start time |  |
| End time |  |
| Interview location |  |
| Interviewer initials | D.D. |
| Transcriber |  |
| Impairment type |  |
| Assistive device |  |
| Yeas lived with disability |  |
| Age |  |
| Sex |  |
| Education level |  |
| Marital status |  |
| Religion |  |
| Employment status |  |
| Profession type |  |
| Income |  |
| Workplace |  |
| Sub-city |  |
| Health Insurance |  |
| Cadre |  |
| General observations (e.g., anything that might impact how the interview is conducted): | |

**Part II: Personal and Daily Life Context**

1. Can you describe your daily household routine and daily life activities?

**Probes:** What do you do during the day? ̶ formal or informal employment, education / study, or other activities)

1. Would you explain how does your condition impact your ability to perform daily activities/ life?

**Probe:**

- Do you need assistance or tools to complete tasks? If yes, what kind?

1. What challenges do you face as a result of your disability in your everyday life?

**Probes:**

- - Are these challenges primarily physical, emotional, or social?
  - How do these challenges affect your health or healthcare needs?

1. How often do you think about your health in relation to your daily activities?

**Probes:**

- - Are there specific activities that make you more aware of your health condition?

1. How confident do you feel in managing your own health, and what kinds of support would improve that confidence?

**Probes:**

- - Are there specific areas where you feel less confident?
  - What tools, resources, or information would help you feel more in control?

1. Who do you live with?

**Probe:** for details on relatives/ household members

**Part III: Understanding Disability and Assistive Devices**

1. Could you tell me about your disability or health difficulties?

**Probes:**

- - The nature of the disability
  - When it was acquired?
  - How it was acquired?

1. Can you tell me you ever been prescribed or used assistive devices to help with your condition?

**Probes:**

- What types of devices or tools have you used? (e.g., wheelchairs, hearing aids, visual aids)
- Were these devices recommended by a healthcare provider, family, or friends?
- How often do you use these devices in your daily life?
- Did you face any barriers in accessing these devices? (e.g., cost, availability)
- Who support for obtaining/ using assistive devices, what kinds of support to help you use or maintain your assistive devices?

1. How well do you feel your assistive devices meet your needs?

**Probes:**

- - Are there any limitations or issues with the devices you use?
  - Do you feel comfortable using them in public spaces or during healthcare visits?

1. Have you experienced any issues with the maintenance or replacement of your assistive devices?
2. How do you feel assistive devices are viewed in your community or by healthcare providers?
3. What improvements could be made in terms of providing access to AT for people with disabilities?

**Probes:**

- - Would suggest in terms of affordability, availability, or any support?
  - What role could healthcare providers/ policymakers play in making AT more accessible?

1. Who helps you with daily activities if needed?

**Probe:** partner involvement, family support

**Part IV: Health status & healthcare accessibility issues**

1. Do you have any health problems (general health condition) that affect your daily life, work, or study?

**Probes:**

- Main health issues you are currently dealing with?
  - Related directly to your disability, or are they separate?
  - How do these health issues affect your daily life & and actions taken?

1. Have you received a formal diagnosis for your health condition?

**Probes:**

- If so, can you describe a recent experience when you sought healthcare?
  - Was the diagnosis process straightforward, or did you face challenges? (at the facility, staff interaction, decision-making, and treatment received)

1. If you did not seek healthcare, can you explain why?

**Probes:**

- - Are there any barriers: financial, social, cultural or medical that limit your access to healthcare?
  - Have you had trouble finding healthcare providers who understand your condition?
  - Reasons and any past negative experiences with clinics or hospitals, psychological factors, such as fear/ anxiety

1. Would you describe your overall access to healthcare services in your community?

**Probes**:

- What are the most significant challenges you face?
- How do these barriers affect your ability to receive care?

1. How do you usually travel to healthcare facilities?

**Probes**:

- What transportation challenges do you encounter, and how do they influence your healthcare access?

1. How do the costs of healthcare services, medications, and transportation affect your ability to seek care?

**Probes**:

- Have financial issues ever prevented you from accessing needed healthcare?

1. Are the healthcare services you require readily available in your area?

**Probe**:

- Have you experienced situations where services were unavailable or delayed, and what impact did that have?
- Is there over appointment because you have a disability?

1. What is the knowledge, attitudes/perceptions & practice of healthcare workers towards persons with disabilities?
2. How do you communicate with healthcare providers?

**Probes**:

- What challenges do you face when trying to explain your health condition to others, especially healthcare providers?
- What measures were taken to resolve them?

1. How do healthcare providers assess and address your health issues during your visits?

**Probes:**

- - Do they seem knowledgeable about your condition?
  - Are you satisfied with the care and attention you receive?

1. How do healthcare providers in your community respond to the cultural and social needs of people with disabilities?
2. How do social and cultural norms in your community affect your ability to access healthcare?

**Probe**:

- Are there any specific norms that create barriers or facilitate access for you?

1. Have you ever experienced stigma or discrimination from healthcare providers or the community due to your disability?

**Probe**:

- How has this affected your healthcare-seeking behaviour?

1. Generally, would you explain about providers either facilitating or hindering your access to care?
2. How do community members know/ aware about, perceive person with disabilities accessing healthcare?
3. What role do your family and friends play in helping you access healthcare?

**Probe**: How important is their support in ensuring you receive the care you need?

1. Are there any local organizations, disability groups, or social services that assist you in accessing healthcare?

**Probe**: How effective are these supports?

1. Are you aware of any government policies or programs designed to assist people with disabilities in accessing healthcare?

**Probe**: How have these impacted your ability to receive care?

1. How would you rate the quality of the healthcare services you receive?

**Probe**: Do you feel that your specific healthcare needs as a person with a disability are fully addressed? or are there any unmet or untreated healthcare needs?

1. How accessible are specialized healthcare services, such as physical therapy, mental health services, or assistive devices?

**Probe**: What challenges do you face in obtaining these services?

1. How accessible is health information for you?

**Probes**:

- Can you easily obtain and understand information about your health and available services?
- How have these barriers been addressed, if at all?
- Disability can be recoded in medical log book?

1. How accessible are public health campaigns and programs for people with disabilities?

**Probes**: Do you feel included in these initiatives?

1. How accessible are health extension packages and programs for people with disabilities?
2. How confident are you in managing your health condition on your own?

**Probes:**

- - Do you feel like you have enough knowledge and resources to manage your condition?
  - What additional support would improve your ability to manage your health?

1. What strategies have you developed to overcome the barriers you face in accessing healthcare?
2. What could be done to improve your experience of healthcare?

**Probe**: Awareness, attitudes, information, availability of services, type of services, supplies/equipment, accessibility, infrastructure, transportation, affordability, cultural values, habits of providers, accommodation of disability, healthcare financing, quality of service, monitoring & evaluation, decision-making, and empowerment

1. Is there anything else you would like to add before we finish? Any comments or questions?

////////////////////////////////// Thank you for participant!!! //////////////////////////
