# Supplementary material for: Left behind in primary healthcare: A qualitative exploration of healthcare experiences of people with disabilities in Ethiopia
Source: PLOS Glob Public Health. 2025 Sep 26;5(9):e0005147. doi: 10.1371/journal.pgph.0005147 (PMC12469150; doi:10.1371/journal.pgph.0005147)
Supplement: S1 Table — (DOCX) [file pgph.0005147.s002.docx]

**Paper-3 (barriers facilitators and suggested solutions)**

**Codebook**

| **Code/ Nodes Name** | **Description** |
| --- | --- |
| (RQ1) Barriers to access healthcare | 1. What are the barriers for accessing healthcare services in disabled people in Bahir Dar City, Ethiopia? |
| Absence of specialized healthcare | Lack of accessible, specialized healthcare services for disabled people. |
| Non-inclusive health information | Absence of disability targeted health information recording, collection, dissemination and use. |
| Communication | Barriers that prevent effective information exchange between healthcare providers and PWDs |
| Anxiety | Expressions of fear/ anxiety about accessing healthcare due to negative outcomes or discrimination. |
| Financial barriers | Economic challenges that restricts ability to afford the direct and indirect cost of healthcare. |
| Inaccessible healthcare facilities | Barriers in healthcare facilities, like absence of ramps, elevators, interpreters, or proper signage. |
| Lack of disability knowledge among HWs | Healthcare providers lacking adequate knowledge to be disability inclusive. |
| Lack of disability training for HWs | Healthcare professionals lacking adequate training to be disability-inclusive. |
| Lack of family and community support | Limited support from family and community networks in helping disabled people access inclusive care. |
| Lack of inclusive outreach health services | Lack of disability targeted community-based health promotion, screenings, & interventions campaigns. |
| Long waiting times in HCFs | People with disabilities face significant and prolonged waiting time for care at public healthcare facilities. |
| Negative attitudes of HWs | Healthcare staff lacking a favourable attitude toward disability inclusion e.g. discrimination. |
| Negative societal attitudes | Any discriminatory remarks and behaviour exhibited by the society towards persons with disabilities. |
| Perceived lack of susceptibility | Individuals with disabilities perceive themselves as less vulnerable to illness or dismiss minor health issues. |
| Policy & implementation gaps | The absence of visible disability-inclusive health policies and its implementation. |
| Poor quality healthcare service delivery | Inadequate and ineffective provision of healthcare services for disability-related pain and complications. |
| (RQ2) Facilitators to access healthcare | 2. What are the facilitators for accessing healthcare among disabled people in Bahir Dar City, Ethiopia? |
| Access to AT in public healthcare facilities | Availability and provision of assistive technology within public healthcare settings. |
| Accessible HCFs | Some facilities have made accessibility improvements (ramps, toilets, doorways). |
| Adherence | Consistent compliance with physician's advice & treatment recommendations. |
| Affordability of healthcare & AT | The financial accessibility of healthcare services and AT for individuals with disabilities. |
| Proximity to healthcare facility | The presence of healthcare facilities within a reasonable short distance |
| Communication skills | Effective communication & self-advocacy skills empower disabled individuals to be disability inclusive. |
| Family and community support | Financial, emotional and practical support from family & community, including help accessing healthcare. |
| Confidence | Self-empowerment and focusing on abilities , a sense of self-assurance |
| Disability awareness among HWs | Most health professionals are knowledgeable about people with disabilities and how to manage their care. |
| Disability training for HWs | Availability of training packages for healthcare workers on disability-specific needs. |
| Drug & supplies | Medications are generally available at the public healthcare facility. |
| Government & policy | Disability mainstreaming is included in the policy & planning documents of government. |
| Health insurance | Family health insurance that provides financial coverage for healthcare expenses of disabled people. |
| Education and knowledge among PWDs | Level of literacy & knowledge about disability right, available services and inclusive care. |
| Lived experiences | Past illness and incidents improved understanding of healthcare and increased the demand for more care. |
| Media coverage of disability awareness | The use of media platforms to promote and raise awareness about disability inclusion. |
| Positive attitude of community members | A positive shift in community attitudes toward disability, driven by religious teachings and awareness. |
| Positive attitude of healthcare workers | A healthcare professional who respects, empathizes with, and is dedicated to attending and treating disabled patients. |
| Reasonable accommodation | Necessary adjustments that ensure people with disabilities have equal access to healthcare services. |
| Religious support | Guidance provided by religious groups or beliefs to help disabled persons seek healthcare services |
| **(RQ3) Solutions to access healthcare** | **3. What are the proposed solutions to improve healthcare access for disabled persons in Bahir Dar City** |
| Addressing negative perceptions | Changing harmful stereotypes or misconceptions towards disability held by communities. |
| Enhance adequate budget allocation | Healthcare facilities should receive sufficient funding to address disability-related & accessibility needs |
| Assign personal assistance in HCFs | Recruitment of personal assistance services for consistent disability support at time of healthcare use. |
| Promoting self-care and prevention | People with disabilities can take proactive steps to maintain their health using available resources. |
| Develop disability-inclusive policy & M&E | The need for effective health policies & its monitoring and evaluation in addressing disability inclusive care |
| Enhance HW disability awareness & training | Improving healthcare workers' awareness and skills through training on disability inclusion and care. |
| Establish sector-wide disability units | Establishing dedicated units for disability affairs in every office or sectors |
| Improve medication and drug supply | Enhancing availability & accessibility of medications and pharmaceuticals to meet healthcare needs. |
| Improving access to assistive technology | Increasing affordability and availability of assistive technology for people with disabilities |
| Improving accessibility of HCFs | Enhancing the physical, financial, and service-related ease of access to HCFs for disabled people. |
| Increase media awareness campaigns | Expanding efforts to raise public awareness of disability inclusion through media campaigns. |
| Conduct research to inform policy | Conduct research in identifying health policy gaps & promoting disability inclusion through policy design. |
| Disability associations and partnerships. | Supporting disability organizations and stakeholder collaboration to advocate for and support disability. |
| Enhance family support | Improving the care, and involvement of family members in supporting disabled people. |
