# Supplementary material for: Left behind in primary healthcare: A qualitative exploration of healthcare experiences of people with disabilities in Ethiopia
Source: PLOS Glob Public Health. 2025 Sep 26;5(9):e0005147. doi: 10.1371/journal.pgph.0005147 (PMC12469150; doi:10.1371/journal.pgph.0005147)
Supplement: S2 Table — (DOCX) [file pgph.0005147.s003.docx]

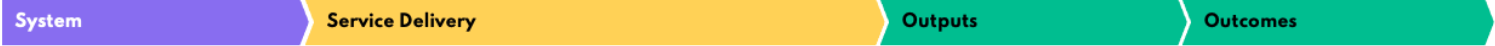


| **Governance** | **Themes** | | **Barriers** | **Facilitators** | **Coping strategies** |
| --- | --- | --- | --- | --- | --- |
|  | **Demand** | **Autonomy & awareness** | Lack of health literacy of communities | Health literacy | Public education & inclusion advocacy |
|  |  |  | Awkwardness/ anxiety | Confidence | Media awareness campaigns |
|  |  |  | Lack of support | Lived experience | Addressing attitudinal barriers |
|  |  |  | Perceived lack of susceptibility | Family & community support | Promoting self-care and prevention |
|  |  |  |  | Acceptance of disability status/ assertiveness | Advocate for nothing about us without us |
|  |  |  |  | Strong in spirituality | Empowerment and involvement in own leadership |
|  |  | **Affordability** | Financial challenges | Health insurance initiation | Establishment of a community fund |
|  |  |  | Lack of health insurance | Financial accessibility | Enhance inclusive budget allocation and healthcare financing |
| **Leadership** |  |  | Budget shortage for inclusivity | NGOs involvement in support | Village based funding |
|  |  |  | Fear of financial loss |  | Increase insurance coverage |
|  |  |  | High cost of healthcare & drugs | Financial support to have AT | Provide free specialized healthcare |
|  |  |  | High indirect cost e.g. transportation |  |  |
|  |  |  | Lack of accommodations for disability-related costs |  |  |
|  |  |  | Low income |  |  |
|  |  |  | Reliance on international donors for AT & healthcare use |  |  |
| **Health Financing** | **Supply** | **Work farce** | Lack of disability knowledge & training | Workforce competency | Disability training for healthcare workers |
|  |  |  | Lack of disability training | Initiation of disability training for HW | Assigning personal assistance in healthcare facilities |
|  |  |  | Negative attitudes of HWs | Favourable attitude | Enhance family support |
|  |  |  | Poor quality of care | Improved communication | Designate a focal person in healthcare facilities |
|  |  |  | Lack of reasonable accommodation | Reasonable adjustment/ prioritization | Sign language training |
|  |  |  | Breach of confidentiality | Improvement in KAP of HWs | Provision of accessible information & communication |
|  |  |  | Non participatory decision making in medication |  | Strengthening social Support Networks |
|  |  |  | Lack of assistance |  |  |
|  |  |  | Lack of family/ community support |  |  |
|  |  |  | Lack of sign language skill |  |  |
|  |  |  | Incomprehensible medical jargon/ derogatory languages |  |  |
|  |  |  | Negative attitudes of society |  |  |
|  |  | **Healthcare facilities** | Distant HCFs | Use of written communication or lip reading | Improve medication and drug supply |
|  |  |  | Inaccessible beds/ examination rooms | Multiple healthcare facility options in Bahir Dar City | Systemic integration of disability issues in health system |
|  |  |  | Inaccessible facilities | Availability of services | Regular facility audits/ assessments |
|  |  |  | Transport challenge | Accessibility of facility | Enhance information accessibility |
|  |  |  | Poor signage & layout of HCFs & units | Accessible health information | Establish disability disaggregated data & evidences |
| **Data & Evidence** |  |  | Long waiting time | Sign language interpreter recruitment by NGOs | Involvement in facility construction |
|  |  |  | Inaccessible health information | Initiation of reasonable accommodation | Advocacy for accessible transportation services |
|  |  |  | Lack of inclusive outreach health services | Availability of drug & supplies | Action to enhance accessibility |
|  |  |  | Lack of disability-specific healthcare information |  | Advocating inclusive healthcare service delivery |
|  |  |  |  |  | Enhance inclusive health data collection system |
|  |  |  |  |  | Ensuring supply of medical equipment and medication |
|  |  |  |  |  | Ensuring accessible health information and reasonable adjustment |
|  |  |  |  |  | Establishing disability units and inclusive governance structures |
|  |  | **Specialized & AT** | Lack of disability-specific healthcare | Accessibility of AT in some public facilities | Initiation of specialized services |
|  |  |  | Insufficient rehabilitation service coverage | Availability of one rehabilitation centre | Initiating local manufacturing of AT |
|  |  |  | Limited physiotherapy facilities |  | Improve availability and affordability of AT |
|  |  |  | Limited supply to AT |  |  |
