# Supplementary material for: Left behind in primary healthcare: A qualitative exploration of healthcare experiences of people with disabilities in Ethiopia
Source: PLOS Glob Public Health. 2025 Sep 26;5(9):e0005147. doi: 10.1371/journal.pgph.0005147 (PMC12469150; doi:10.1371/journal.pgph.0005147)
Supplement: Attachment — Submitted filename: Response to Reviewers.docx [file pgph.0005147.s005.docx]

**Authors Response to Reviewers**

**Overall Assessment and Recommendation**

1. **Comments:**  This manuscript addresses a critical and underrepresented issue in global health—the healthcare experiences of people with disabilities in Ethiopia—and offers rich, participant-centred insights grounded in a rights-based framework. The topic is timely, the methodology is generally sound, and the findings have strong potential to inform policy and practice. However, the manuscript would benefit from clearer articulation of its unique contribution to existing literature, a more precise methodological framing, improved findings and discussion sections, and discussion of limitations outlined below. With thoughtful revision, this study has the potential to make meaningful contribution to PLOS Global Public Health and to the broader discourse on disability-inclusive health systems.

**Response:** Thank you for your thoughtful summary and comments. The manuscript now more clearly highlights its distinct contribution to literature, particularly its focus on disability-inclusive health in a low-income African context using a rights-based lens. The methods section has been revised to better explain the study design, sampling, and analytical approach. We have revised the findings section to increase clarity, with strong thematic organization and integration of participant voices. The discussion more directly links results to existing literature and policy implications. Lastly, this revision also added to acknowledge study limitations and researcher positionality.

**Introduction:**

2. **Comments:** The introduction section is well-structured with appropriate references to disability rights and access literature. However, it would have benefited from employing disability justice or feminist disability studies to better surface the intersectional nature of the barriers. This would have enhanced the global relevance of the argument raised in the manuscripts and move it beyond descriptive public health framing.

**Response:** Thank you! We incorporated evidence from disability justice, gender and disability studies. Specifically, we introduced intersectionality as an overlapping system of discriminations rooted from gender, disability, race, socioeconomic status etc and the importance of recognizing disability as a structurally determined identity rather than merely an individual impairment and medical condition or service-delivery issue. This is by focusing how structural inequities—linked to sexual and gender minorities, poverty, and social marginalization—shape healthcare access for people with disabilities which conceptualize disability as a socio-political identity intertwined with race, gender, and class, rather than a remarkable health outcome alone. Page 3, Paragraph 3 (Lines 77–85).

**Theoretical Framework:**

3. **Comments:** The Missing Billion framework seems to be an appropriate one for applied health systems analysis and disability-inclusive design. Yet the framework is not rooted in qualitative approach or phenomenology. So: If the goal is to inductively indicate lived experience, the framework should not be used to the code qualitative data directly. It should be introduced in the Methods as a secondary analytic or interpretive tool, not as a deductive framework.

**Response:** Thank you!. The methods section was revised to clarify that the primary qualitative analysis followed a deductive coding & thematic analysis to capture participants’ experiences in accessing healthcare (Page 8, line:198-203). A predefined codebook— was developed from piloted test transcriptions, the interview guide tool, and using the farmwork —guided the coding process then the analysis. We have clarified this in the text on page 8, line number 198 - 206.

4. **Comments:** I recommend the authors clarify how and when the framework was used in the research process. Was it applied after thematically analyse data, to interpret emergent findings, or did it guide interview design and initial coding? If the study is primarily inductive and experience-focused, the framework should be presented as a secondary interpretive lens. This distinction would improve methodological transparency and preserve the integrity of the qualitative approach.

**Response:** Thank you for your comment. We have clarified the methods section to specify the role and timing of the framework in the research process. The primary analysis followed deductive coding and then thematic approach (Page 8, line:198-206) not inductive one to explore participants’ lived experiences in accessing healthcare. The interview design or initial coding was developed in reference to the Missing Billion framework. Also, the predefined codebook or coding framework—developed from the interview guide, pilot data, based on the framework—which further guided the coding process.

**Methods:**

5. **Comments:**  The authors indicated that the study is “phenomenological,” yet the analysis follows reflexive thematic analysis. Consider rephrasing this as a qualitative exploratory study informed by phenomenological approach, rather than claiming a strict phenomenological design—since phenomenological analysis techniques such as bracketing, horizontalization, textural and structural synthesis, and essence formulation—are not clearly employed or reported.

**Response:** We have revised the manuscript to describe the study as a qualitative exploratory study design. Page number: 6, line number:145 -149

6. **Comments:** I suggest the authors either:

a) Clarify and justify their use of a phenomenological method by explicitly outlining steps taken (e.g., bracketing, meaning-making, synthesis), or

b) Reframe the methodology as a qualitative thematic study informed by phenomenological sensitivity, rather than a strict phenomenological design. This would better reflect the analytic procedures and strengthen the study’s methodological coherence.

**Response:** Thank you! We have revised the manuscript to describe the study as an qualitative study using thematic analysis instead of presenting it as a pure phenomenological design. Page number: 6, line number: 145 -149

7. **Comments**: The manuscript appropriately highlights the lead researcher's positionality as a person with a disability, which likely facilitated trust and empathetic engagement with participants. However, given the gendered dimensions of healthcare access discussed in the findings—particularly by women with disabilities—reflecting on how gendered power relations influence data collection, rapport, and interpretation, would have strengthened the pieces on reflexivity.

**Response:** Thank you! We acknowledge that being a male interviewer could affect the depth of disclosures on gender-sensitive topics, such as reproductive or maternity health. However, during interviews, we did not observe clear discomfort or avoidance of mentioning gender sensitive health topics (e.g. maternity services, reproductive including abortion services etc). This might be due that we ensured a comfortable interview environment, allowed participants to choose the location, and emphasized voluntary disclosure, confidentiality, and the right to skip or decline questions. Although no participant declined to respond to such a question, it is possible that some disclosures were shaped by the researcher’s gender. This dynamic was carefully considered during interpretation of the data and is acknowledged as a limitation in the study as well. We now discuss this as limitation and a consideration for future studies, where matching participant and interviewer gender might enhance disclosure on sensitive issues. Page number 8, line number 184 – 188; page number: 22; line number: 617 **–** 619.

**Results:**

8. **Comments:** The presentation of barriers, facilitators, and solutions is rich and logically structured. The manuscript includes references to percentages when describing the background of participants. I recommend avoiding quantitative expressions in this context, as they can mislead readers into interpreting the data as statistically representative. It will be better to focus on variation than aggregation. Instead, consider using qualitative descriptors such as “several,” “many,” or “a few.

**Response:** We have revised the manuscript to replace quantitative expressions (e.g., percentages) with qualitative descriptors such as ‘most’’, ‘’some’’, “many,” or “a few,” as suggested. Page #: 10; line #: 246- 256

9. **Comments:** On Beliefs in Incurability as a Barrier: The manuscript frames beliefs around the “incurability” of disability as a barrier to care. However, it is essential to distinguish between curability and treatability in the context of disability. Many disabilities are indeed not curable but can be effectively managed with appropriate treatment methods. Therefore, framing the belief in incurability as “misinformation” could undermine realistic understandings of chronic disability conditions. I recommend the authors reframe this point to recognize that while biomedical cures may not exist for all disabilities, this does not equate to hopelessness or justify neglect in care.

**Response:** Thank you for this insightful and important clarification. We fully agree that the distinction between curability and treatability is critical in discussions of disability and healthcare access. As a misinformation, some communities, due to cultural beliefs perceive disability as permanent, which may lead to avoidance of care or hopelessness. However

in the context of this study, our intention was not to suggest that all disabilities are curable or incurable, but rather to explore how prevailing community beliefs about disability and healthcare access can contribute to therapeutic challenges in care. These beliefs—whether based on misinformation or genuine perception—carefully examined through our interviews, transcription, translation and analysis. So, in the context of disability, we believe that most disabilities are treatable in ways that improve function, health, and quality of life through rehabilitation, assistive devices, therapy, or inclusive support. This perspective aligns more closely with the social and rights-based models of disability, which emphasize participation and support, rather than the medical model, which frames disability primarily as a problem to be fixed. Thus, we agree that emphasizing curability—or framing disability solely in terms of incurability—can lead to therapeutic hopelessness or neglect in care. (i.e., giving up on care) when a cure is not possible. Because treatability emphasizes ongoing care, inclusion, and intervention, we have revised the manuscript carefully to reflect this distinction. We also took great care to review again the transcription verses translation of our raw audio data into English, ensuring that our transcripts were accurate. Page number: 10, Line number: 265 – 270

10. **Comments:** After finishing reading the manuscript, I have still confused about the scope of the healthcare access the authors explored. Several participant quotes indicate both general and disability-specific healthcare, making it difficult to clearly delineate the scope of services under discussion. For example, quotes about drug stockouts, insurance exclusion, or religious interpretations of disability are not consistently framed as applying to all health needs or only to disability-specific care. Clarifying this distinction particularly in the introduction and methods—would help the reader interpret whether the reported barriers reflect exclusion from the broader healthcare system or inadequacies in disability-targeted care. This distinction also has implications for policy and interventions.

**Response:** We have revised the introduction and methods sections to clarify that the study explored access to both general healthcare services that is broader healthcare system and disability-related/ targeted care, as experienced and understood by participants. This includes routine health services/ general (e.g., outpatient visits, ANC services, vaccination, medical check ups, insurance coverage) as well as services more directly related to disability needs (e.g., mental health, rehabilitation, assistive technology, communication accommodations — e.g. sign language, braille). Generally, Theme V—Rehabilitation, Specialized Services, and Assistive Technology—clearly reflects disability-related service accessibility. In the remaining themes, both general or mainstream services and disability-related aspects are discussed. Page number: 3, Line number: 62 – 73; page number: 4, line number, 90- 93; Page number: 6; line number:146 – 148; page 9, line number 223 – 225; page number 11, line number 296 & 307, page number 23, line number 626 - 629

11. **Comments:** Given the phenomenological and retrospective nature of the in-depth interviews, there is a potential for recall bias, particularly as some participants reflected on healthcare experiences that occurred many years ago—including during childhood. While the study emphasizes rich narrative accounts, it would strengthen the methodological rigor to explicitly acknowledge the possibility of recall bias as a limitation. Additionally, it may be helpful for the authors to clarify how they addressed this in data collection or analysis (e.g., triangulation, probing recent events, or reflective journaling by the interviewer). (Page 13 for instance. Please consider adding line numbers in future manuscript versions to support more precise referencing and constructive feedback during peer review).

**Response:** Thank you for this insightful comment. We agree the retrospective nature of in-depth interviews may introduce the potential for recall bias, particularly when participants recount healthcare experiences from earlier periods of their lives, including childhood. So, we have now explicitly acknowledged recall bias as a potential limitation in the revised manuscript, limitation section (page: 8, line number: 185 – 188, Page number 9; line number: 225 - 231, page number: 22; line number: 614 - 616). In addition, we have elaborated on the strategies used during data collection and analysis to mitigate its impact. These included: probing through follow up questions for recent and specific experiences during interviews to help anchor participant narratives in time and enhance recall accuracy; and the use of reflective journaling (On page # 9, line number:229 -231; page: 9; line number: 225 - 227. Credibility was enhanced through prolonged engagement and the application of set aside (on page 8/9, line number 213 - 215). The line number was added to the revised manuscript to facilitate referencing.

**Discussion:**

12. **Comments:** The discussion offers important interpretations grounded in lived experiences of people with disabilities. However, I recommend the authors reflect more critically on the limitations of their study to enhance transparency and contextual integrity. While I understand that design elements cannot be altered at this juncture, these limitations can be acknowledged and leveraged to inform the interpretation of findings and shape future research.

**Response:** We have revised the discussion particularly limitation section to provide a more explicit and nuanced reflection on key limitations of the study, example the retrospective nature of the data, the potential for recall and social desirability bias, the limited geographic scope, and the reliance on participants’ self-reported experiences without direct observation. We have also clarified how these limitations may influence the interpretation of findings and have suggested ways future research could build on and address them—for example, through longitudinal designs, broader sampling strategies, or mixed-methods /approaches. We agree that openly acknowledging these limitations strengthens the contextual integrity of the study and can help guide future inquiry and policy engagement in this area. Page number 22/23: line number: 614 – 637.

13. **Comments:** These may include:- Scope Ambiguity: It remains unclear whether participants were referring to general healthcare access (e.g., for non-disability conditions) or specifically to disability-related services (e.g., rehabilitation). Clarifying this distinction—or at least acknowledging the ambiguity—would help readers better assess the applicability of the findings and recommendations.

**Response:** Thank you! We have clarified in the introduction and methods sections that the study examined both general healthcare services and disability-specific/related care, as reported by participants. Where the distinction between general and disability-specific services was unclear, we have acknowledged this ambiguity as a limitation as well. We acknowledge that, in some instances, participant narratives did not clearly distinguish between general healthcare access and disability-specific services . So, we have revised the manuscript to more clearly acknowledge this ambiguity in the introduction, methods and discussion sections. Page number: 3, Line number: 60 – 63; page number: 4, line number, 90 - 95; Page number: 6; line number:146 – 148 ; Page number: 23, line number:626 – 628.

14. **Comments:** Recall Bias: Several participants shared experiences from early life, sometimes decades ago. This introduces the risk of recall bias, particularly for emotionally charged or stigmatizing events. While this is a common challenge in qualitative research, acknowledging it would enhance the methodological transparency.

**Response:** Thank you! We agree that the inclusion of long-term retrospective accounts, particularly those involving emotionally significant or stigmatizing events, introduces the potential for recall bias—a known limitation in qualitative research of this nature. Thus, we have revised the limitations section to explicitly acknowledge the possibility of recall bias and its implications for the accuracy and completeness of participant narratives. We have also clarified that while such bias cannot be entirely eliminated, we sought to mitigate it during data collection through careful probing, use of contextual cues, and by encouraging participants to share concrete and detailed experiences where possible. (page: 22, line number: 615 – 617; page: 8, line number: 185 - 188; page; page: 9, line number 225 - 231).

15. **Comments:** Lack of Triangulation with Provider or Policy Perspectives: Including insights from healthcare workers or administrators could have enriched the analysis, especially regarding health system constraints and policy feasibility. I suggest acknowledging this as a limitation and a direction for future work.

**Response:** Thank you! We agree that incorporating the perspectives of healthcare providers or administrators would have added valuable depth to the analysis, particularly in relation to health system constraints, service delivery challenges, and the feasibility of proposed recommendations. We have now acknowledged this as a limitation in the revised manuscript section and have identified it as an important direction for future research. Page numbers: 23; line number: 634 - 637.

16. **Comments:**  Framework Fit: While the Missing Billion Framework was well-applied, the manuscript could more critically discuss its limitations—particularly around overlapping categories (e.g., affordability vs. financing) and potential blind spots regarding intersectional factors (gender, poverty, urban ways of life). Addressing this would show a deeper engagement with the chosen analytical lens.

**Response:** Thank you**!**. We acknowledge in the limitation section that potential blind spots in the framework’s ability to fully capture intersectional influences such as gender, poverty, and urban living conditions—factors that emerged prominently in participants’ narratives. Additionally, conceptual overlaps between domains like affordability and financing may obscure distinct issues. We encourage future studies to adopt strategies that mitigate these limitations for more nuanced analysis. Page number: 23, Line number: 628-632 versus 619 -622.

**Conclusion:**

17. **Comments:** Strong and policy-relevant. However, a final sentence that ties the findings back to specific SDG goals may help reinforce global significance.

**Response:** Thank you!; In the conclusion part, we explicitly links the study's findings to relevant Sustainable Development Goals (SDGs)—particularly SDG 3 (Good Health and Well-being), and SDG 10 (Reduced Inequalities) as explained on page 23/24 line number 655 – 657.
